# Feedback-Related Negativity in Children with Two Subtypes of Attention Deficit Hyperactivity Disorder

**DOI:** 10.1371/journal.pone.0099570

**Published:** 2014-06-16

**Authors:** Jingbo Gong, Jiajin Yuan, Suhong Wang, Lijuan Shi, Xilong Cui, Xuerong Luo

**Affiliations:** 1 Mental Health Institute of The Second Xiangya Hospital, National Technology Institute of Psychiatry, Key Laboratory of Psychiatry and Mental Health of Hunan Province, Central South University, Changsha, Hunan, China; 2 Traditional Chinese Medicine University of Hunan, Changsha, Hunan, PR China; 3 School of Psychology, Southwest University, Chongqing, China; 4 Department of Neuroscience, the Third Affiliated Hospital of Soochow University, Changzhou, China; University Children's Hospital Tuebingen, Germany

## Abstract

**Objective:**

The current model of ADHD suggests abnormal reward and punishment sensitivity, although differences in ADHD subgroups are unclear. This study aimed to investigate the effect of feedback valence (reward or punishment) and punishment magnitude (small or large) on Feedback-Related Negativity (FRN) and Late Positive Potential (LPP) in two subtypes of ADHD (ADHD-C and ADHD-I) compared to typically developing children (TD) during a children's gambling task.

**Methods:**

Children with ADHD-C (n = 16), children with ADHD-I (n = 15) and typically developing children (n = 15) performed a children's gambling task under three feedback conditions: large losses, small losses and gains. FRN and LPP components in brain potentials were recorded and analyzed.

**Results:**

In TD children and children with ADHD-C, large loss feedback evoked more negative FRN amplitudes than small loss feedback, suggesting that brain sensitivity to the punishment and its magnitude is not impaired in children with ADHD-C. In contrast to these two groups, the FRN effect was absent in children with ADHD-I. The LPP amplitudes were larger in children with ADHD-C in comparison with those with ADHD-I, regardless of feedback valence and magnitude.

**Conclusion:**

Children with ADHD-C exhibit intact brain sensitivity to punishment similar to TD children. In contrast, children with ADHD-I are significantly impaired in neural sensitivity to the feedback stimuli and in particular, to punishment, compared to TD and ADHD-C children. Thus, FRN, rather than LPP, is a reliable index of the difference in reward and punishment sensitivity across different ADHD-subcategories.

## Introduction

Attention deficit hyperactivity disorder (ADHD), which is the most prevalent childhood neuropsychiatric disorder, is defined in the current Diagnostic and Statistical Manual of Mental Disorders (DSM-IV-TR) as developmentally inappropriate and impairing symptoms of inattention and/or hyperactivity-impulsivity [1]. ADHD affects 5.29% of school-age children worldwide [2], 7.8% in the USA [3] and 7.5% in Taiwan [4]. According to the DSM-IV, ADHD has three subtypes: predominantly inattentive type (ADHD-I), which accounts for 45% of all ADHD patients; predominantly hyperactive-impulsive (ADHD-H), which affects 21% of all patients; and combined-type (ADHD-C), which occurs in 34% of all patients [1], [5].

Recent studies imply that ADHD is associated with an aberrant sensitivity to reinforcement [6]–[11], such as reward and punishment. Since reinforcement is highly associated with motivation, evidence suggests that an unusually low level of effort or intrinsic motivation accounts for the performance deficits in children with ADHD [12]–[15]. Behavioral studies have demonstrated that children with ADHD show an abnormal sensitivity to motivational cues. They have problems in maintaining optimal performance when they have to rely solely on their intrinsic motivation, i.e. without external motivators such as feedback or reward [16], [17]. Furthermore, children with ADHD show an attention span that is very limited without supervision or when tasks are extremely boring [15], but benefit to a greater extent from reinforcement contingencies implemented in a task [18]. Motivational models of ADHD formulated several different predictions about reinforcement sensitivity (1) a preference for small immediate reward over larger delayed reward [19]–[21]; (2) reduced neurobiological sensitivity to reward [19], [22] and reduced reward anticipation [20], [21], and (3) reduced behavioral sensitivity to cues of aversive stimuli in general [23], [24]. In addition, from a clinical perspective, reinforcement contingencies are found to normalize behaviors that characterize ADHD [25]–[27]. However, other studies have predicted that individuals with ADHD have increased sensitivity to punishment [28], [29]. Recently, several event-related potential studies investigated how reward and punishment influences the monitoring of performance feedback in children with ADHD. van Meel et al. [30] reported that the FRN amplitude to losses was more pronounced in the ADHD group, suggesting an enhanced sensitivity to unfavorable outcomes in children with ADHD. However, in another study by van Meel et al. [31], the FRN effect was found to be entirely absent in children with ADHD. The lack of modulation of the FRN by contingencies in ADHD suggests deficient detection of environmental cues as a function of their motivational significance. Interestingly, the finding is consistent with the report by Groen et al. [32] that the Methylphenidate-free (Mph-free) children with ADHD did not show an FRN effect in the loss condition. This may be indicative of either difficulties in computing reward prediction errors or more positive expectancies of their outcomes in the face of losses. These discrepancies arise, at least in part, from the fact that these studies did not classify ADHD children into different subtypes. However, this classification is important, because there are evidences that different subtypes of ADHD are associated with different reward or punishment processing [33], [34]. To our knowledge, there are no reports describing differences among ADHD subgroups in the monitoring of performance feedback coupled with reward and punishment.

Emotional-motivational dysfunctions are likely to contribute to ADHD, especially to hyperactive and impulsive symptoms. Regarding subgroup differences, ADHD-C and ADHD-HI in particular should show emotional-motivational dysfunction, while ADHD-I is less likely to show differences. A reduced responsiveness of the reward system was found to be associated with hyperactive-impulsive symptoms but not with inattentiveness [33]. In ADHD-C, symptoms of inattentiveness may be a compensatory factor for blunted response to unpleasant stimuli, as these individuals are associated with internalizing behavior [35] marked by high reactivity to unpleasant stimuli. ADHD-C and ADHD-I differ in a variety of ways that could contribute to different patterns of motivational style. Children with ADHD-C are more likely to show comorbid externalizing and impulsive behavior. Moreover, the nature of the cognitive-attentional deficit appears to differ between subtypes, with the ADHD-C group characterized as distractible, sloppy and disorganized and the ADHD-I group characterized as drowsy, sluggish and less alert [36], [37]. Carlson et al. [38] observed some motivational style differences between ADHD subtypes, with the ADHD-C group more motivated by competitiveness and a desire to be perceived as superior to others and the ADHD-I group less uncooperative and possibly more passive in their learning styles. In addition, a recent study [34] showed that healthy subjects exhibited startle response attenuation and potentiation by pleasant and unpleasant pictures, respectively. In ADHD with impulsive and hyperactive symptoms, the startle responses was not attenuated by pleasant and not potentiated by unpleasant stimuli. The startling response in ADHD-I was attenuated to a lesser degree by pleasant stimuli compared to the control group. These findings suggest that blunted emotional reactivity is especially pronounced in ADHD patients with symptoms of hyperactivity and impulsivity (ADHD-C, ADHD-HI). Recently, a fMRI study [39] compared neural activation in the ventral striatum and prefrontal regions during reward processing in ADHD subtypes and healthy adults. The result showed that, compared to ADHD-C and healthy subjects, ADHD-I subjects showed a bilateral ventral striatal deficit during reward anticipation. In contrast, ADHD-C subjects showed orbitofrontal hyporesponsiveness to reward feedback when compared with ADHD-I and healthy subjects.

The late positive potential (LPP) is a central-parietal positive slow ERP that reaches the largest amplitudes at 500–700 ms post-stimulus and lasts for several hundred milliseconds [40]. Specifically, many studies have revealed that the LPP is more pronounced for emotionally salient than for neutral stimuli [41]–[44]. LPP has been described to reflect increased levels of sustained attention to effective-motivational stimuli [45]–[47], its amplitude decreased with the reduction of experienced emotion arousal, and increased with enhancement of the arousal [40]. Making use of a time production task with visual performance feedback, van Meel demonstrated that children with ADHD showed a decreased late positivity (after 450 ms) to negative feedback stimuli indicating loss. This result has been interpreted as a deficit in the affective evaluation of feedback signals and altered evaluation of future consequences in children with ADHD. Furthermore, a study of Groen consistently reported aberrant late processing of non-reward and punishment (increased feedback LPP) in children with ADHD. However, another study by Groen [48] demonstrated a trend toward a reduced LPP in response to negative feedback stimuli in children with ADHD.

Numerous studies have reported that the human brain is especially sensitive to emotionally negative events, and that these events are preferentially processed relative to neutral and positive events [49]–[52]. Behavioral studies [53]–[55] have demonstrated that, relative to positive events, negative events recruit attentional resources more rapidly, or automatically. Moreover, the magnitude of the negative event is important, as extremely negative events typically represent a greater threat to survival than do moderately negative events [56], [57]. Yuan et al. reported that humans are more sensitive to magnitude differences in negative stimuli, and these different magnitudes could be clearly differentiated in each step of information processing stream even when individuals are highly engaged in a non-emotional task [56], [58]. In contrast, magnitude differences in positive events are often unattended to in daily life, as positive events typically bring no harm [56]. In our daily life, more negative stimuli are punishments or losses, not violence or traffic accidents. An issue that has yet to be investigated is whether children with ADHD are also sensitive to punishment and its magnitude, and how ADHD children with different symptoms may differ in this sensitivity.

Therefore, the present study investigated the monitoring of performance feedback coupled with score losses and gains in children with two subtypes of ADHD (ADHD-C and ADHD-I). In order to evaluate the effect of punishment on the monitoring of performance feedback, we manipulated the magnitude of punishment (−1, −4). Regarding the typically developing (TD) children, based on the outcomes of the study of van Meel and colleagues [31], we expected that they would show enhanced monitoring of feedback as reflected by an enhanced FRN to large losses compared with that to small losses and gains. Based on the study by Carlson [38], we predict that patients with ADHD-C, a group characterized by competitive motivation and the desire to be perceived as superior to others, would show more sensitivity to large punishment as reflected by an enhanced FRN to large losses compared with that to small losses. Regarding the children with ADHD-I, who have severe attention problems as the core symptom, we expected that they would show aberrant early feedback detection as reflected by a reduced or absent FRN under all conditions. As previous studies have demonstrated, the LPP may reflect the late processing of the affective value of feedback stimuli in the context of feedback processing [31], [48], [59]. Groen [32] speculated that the children with ADHD attached more value to the feedback stimuli than the TD children, because they are more dependent on external motivators to maintain their performance. Based on this speculation, we predict that children with ADHD-C show an enhanced feedback LPP to larger punishment. As the LPP has been described to reflect increased sustained attention to affective-motivational stimuli and the LPP amplitude depends on how the emotional stimuli are appraised and attended to, we predicted that children with ADHD-I with severe inattention problems would not show LPP effect during feedback processing.

## Methods

### Ethics Statement

This study was approved by the Ethics Committee of Xiangya Second Hospital. Written informed consent was obtained from all the children and their parents. All participants were informed of their right to withdraw from the study at any time.

### Participants

The participants were 31 medication-free (stimulant-naive) children with ADHD (27 boys and 4 girls), aged 6–14 years, who were outpatients at the Clinic of Child Psychiatry of the Second Xiangya Hospital of Central South University. The inclusion criteria were as follows: meeting the DSM-IV-TR criteria and the Kiddie-Sads-Present and Lifetime Version (K-SADS-PL); no history of serious head injury, epilepsy, drug abuse or psychiatric disorders (e.g., schizophrenia, pervasive development disorder, mental retardation or mood disorder). The children with ADHD were classified into two subtypes: ADHD-I (n = 15), ADHD-C (n = 16). Fifteen TD children served as controls. The TD children were selected from local primary schools, and matched well with the ADHD group with respect to age and gender. The K-SADS-PL served to exclude TD children who displayed symptoms of ADHD. All participants reported normal or corrected-to-normal vision and were right-handed. Their full-scale IQ was above 80, according to the Wechsler Intelligence Scale for Children - Revised in China (C-WISC). Table 1 presents the demographic characteristics of the ADHD group and the TD group. There were no significant differences between the groups with respect to sex and mean age.

**Table 1:** Group characteristics (ANOVA test statistics).

Group	TDs (n = 15)	ADHD-C (n = 16)	ADHD-I(n = 15)	F value (ANOVA)	p value (ANOVA)	p value (ADHD-I v.s. ADHD-C)	p value (ADHD-I v.s. TD)	p value (ADHD-C v.s. TD)
Gender (M/F)	11/4	13/3	14/1		ns(Fisher's exact test)			
Age (yrs)	10.47(2.53)	9.50(2.34)	8.87(1.51)	1.878	ns			
IQ score	119.07(12.72)	102.53(11.27)	104.50(15.23)	7.053	0.002	0.639	0.005	0.001
SNAP-IV								
Total score	14.64(2.65)	31.38(6.82)	20.07(3.67)	47.52	<0.001	<0.001	0.03	<0.001
Inattentionitem score	7.47(1.68)	16.56(4.27)	14.67(2.72)	46.83	<0.001	0.095	<0.001	<0.001
Hyperactivity/Impulsive item score	7.00(1.46)	14.81(3.39)	5.40(2.47)	59.20	<0.001	<0.001	0.098	<0.001
Child Behavior Checklist								
total problems	34.99(11.00)	63.22(22.57)	48.01(9.20)	12.66	<0.001	0.01	0.028	<0.001
Inattention item score	3.07(2.49)	8.31(3.55)	6.27(2.87)	11.84	<0.001	0.235	0.009	<0.001
internalizing problems	2.60(1.84)	8.31(5.77)	5.13(3.34)	7.78	0.001	0.034	0.093	<0.001
externalizing problems	3.47(2.70)	16.87(10.56)	10.07(8.90)	13.67	<0.001	0.091	0.001	<0.001

Note: TD = Typically developing, SNAP-IV = The Swanson, Nolan and Pelham Rating Scale; CBCL = The Child Behavior Checklist; IQ = Intelligence Quotient.

The Child Behavior Checklist (CBCL) [60], [61] is a well-studied standardized instrument that asks parents to assess their child's competencies and behavioral and emotional problems on 118 specific items, using a 3-point scale (0 = “not true”, 1 = “somewhat/sometimes true” and 2 = “very/often true”). The clinical scales of the CBCL contain a Total Problems score, two subscale scores (Internalizing and Externalizing Problems) and eight syndrome scales: Aggressive Behavior, Delinquent Behavior, Withdrawn, Somatic Complaints, Anxious/Depressed, Attention Problems, Social Problems and Thought Problems. The presence of psychopathology of TD children was checked by CBCL. None of the TD children scored within the clinical rage of subscales and the total problem scale of CBCL.

The symptoms at each ADHD cluster (inattention, hyperactivity/impulsivity and combined) were obtained from Swanson, Nolan, and Pelham Scale- version IV (SNAP-IV) [62], a widely used standandized measure of ADHD. The SNAP-IV scale has four subscale (Total Scores: 26 items; Inattention: 9 items, and Oppositional: 8 items). Each item is scored for severity on a 4-point scale (0 = “not at all,” 1 = “just a little,” 2 = “quite a bit” and 3 = “very much”). The scale was completed by the participants' parents. Individuals with 6 or more symptoms of inattention but fewer than 6 symptoms of hyper-impulsive were identified as inattentive type, participants with 6 or more hyper-impulsive symptoms and fewer than 6 symptoms of inattention were categorized as Hyper-Impulsive type, and individuals with 6 or more symptoms on both dimensions were identified as combined type.

### Children gambling paradigm

We modified the gambling task according to the children's cognitive characteristics. The experiment was programmed and executed with E-prime (Psychology Software Tools, Pittsburg, PA). The task was administered under three reinforcement-contingency conditions: reward, small punishment and large punishment, which were operationalized as score gains and losses. In each trial, the participants were presented with two “doors” side by side (3000 ms) in the center of a black screen, following a 600–1000 ms central fixation cue. Participants were told that there was a happy face behind one door and a sad face behind the other door. Participants used a response box to make their choices. After an 800 ms interval, the feedback was displayed on the screen (1500 ms) by a number that indicated what the participants gained or lost. The happy face was always “+2” (Reward, RD), while the sad face could be “−1” (Low-deduct, LD) or “-4” (High-deduct, HD). The RD:LD:HD ratio was 6∶2∶2. All stimuli were randomly presented. If the participants did not respond within the required time (3000 ms), while the doors were on the screen, the computer would choose one door at random.

The task took approximately 30 min and consisted of 10 blocks, with each block including 30 trials. The participants had a rest after each block. The children were told that they would be given the final reward according to the all rewards or punishments of the whole task. However, unknown to the children, happy faces and sad faces were randomly presented independent of their response. The children were simply told to “make as many scores as possible.” All subjects were provided with the same amount of compensation, 50 yuan RBM.

### EEG acquisition and analysis

The electroencephalography (EEG) measures were recorded continuously (−200 ms–0 ms; 500-Hz sampling rate; FCZ reference; AFZ ground; BrainAmp amplifiers) from 36 scalp sites using Ag/AgC1 electrodes mounted on an elastic cap, according to the 10–20 international system. The array included 7 midline sites (OZ, POZ, PZ, CPZ, CZ, FCZ, and FZ), 12 sites over each hemisphere (O1/O2, PO3/PO4, PO7/PO8, P3/P4, P7/P8, CP3/CP4, C3//C4, FC3/FC4, FT7/FT8, F3/F4, F7/F8 and FP1/FP2), the left and right mastoids (TP9, TP10), and the horizontal electrooculogram (EOG; FP1) and the vertical EOG (FP2) electrodes to monitor the EOG bipolarly. All electrode impedances were kept below 5 kΩ. The EOG was recorded for the purpose of artifact correction; eye movement and blink artifacts were corrected using the Gratton et al. [63] algorithm. Offline analysis was performed using Brain Vision Analyzer software 2.0 (Brain Products, Munich, Germany). All data were re-referenced to the average reference offline and digitally low-pass-filtered at 30 Hz. Epochs of 1200 ms were extracted from the continuous data file for analysis, commencing 200 ms before the feedback stimulus. Grand-average ERPs were obtained by averaging the data of individual participants according to each condition and feedback type. The average number of trials across the three feedback conditions was not significantly different across the TD, ADHD-I and ADHD-C groups [*F* = 1.000, *P* = 0.371]. The averaged number of trials was 84.49 for TD group,78.98 for ADHD-I group, 70.85 for ADHD-C group. Additionally, there was no significant interaction effect between group and feedback [*F* = 1.943, *P* = 0.156]. The average number of trials in the reward condition was 151.33 for TD group, 133.62 for ADHD-C group, 140.07 for ADHD-I group [*F* = 1.670, *P* = 0.20]. In addition, the averaged number of trials in the small loss condition was 55.80 for TD group,51.53 for ADHD-I group, 48.81 for ADHD-C group [*F* = 2.64, *P* = 0.08]. Lastly, the averaged number of trials during the large loss condition was 46.33 for TD group, 45.33 for ADHD-I group, 43.00 for ADHD-C group [*F* = 0.64, *P* = 0.53].

To assess the ERP waves elicited by feedback stimuli, we measured the average amplitude of FRN (250–350 ms) from Fz to Cz sites and the average amplitude of Lpp (400 ms–800 ms) from Pz site. A three-way ANOVA was conducted on the FRN component. ANOVA factors were feedback type (larger loss, smaller loss and gain feedback), electrode site (Fz, Cz) and group (TD, ADHD-C and ADHD-I participants). The same analysis was conducted for the LPP at Pz. The ERP data were analyzed using Brain Products Analyzer software. The degrees of freedom of the F-ratio were corrected according to the Greenhouse-Geisser method.

## Results

**Figure 1:**
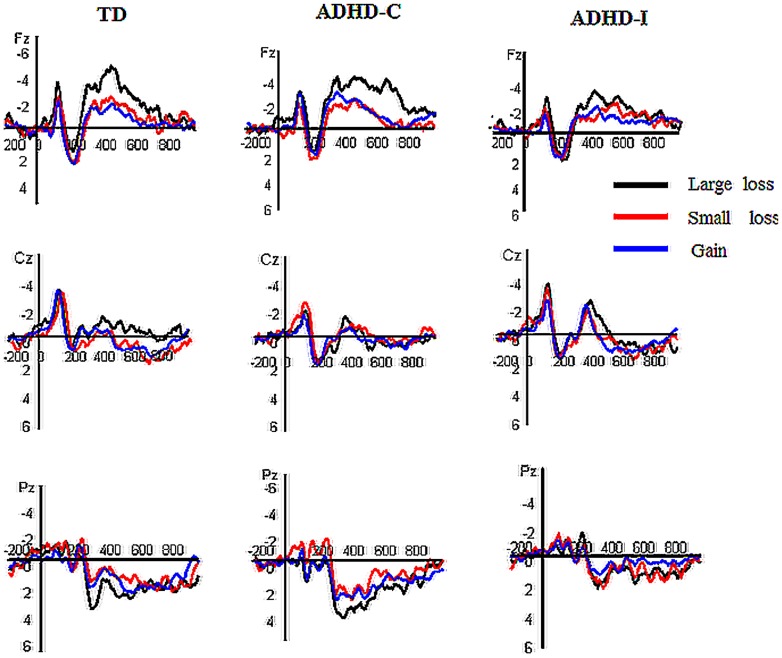
Grand-average of ERPs at Fz, Cz and Pz for the large loss (black lines), small loss (red lines) and gain (blue lines) feedback conditions in the TD, ADHD-C and ADHD-I groups.

**Figure 2:**
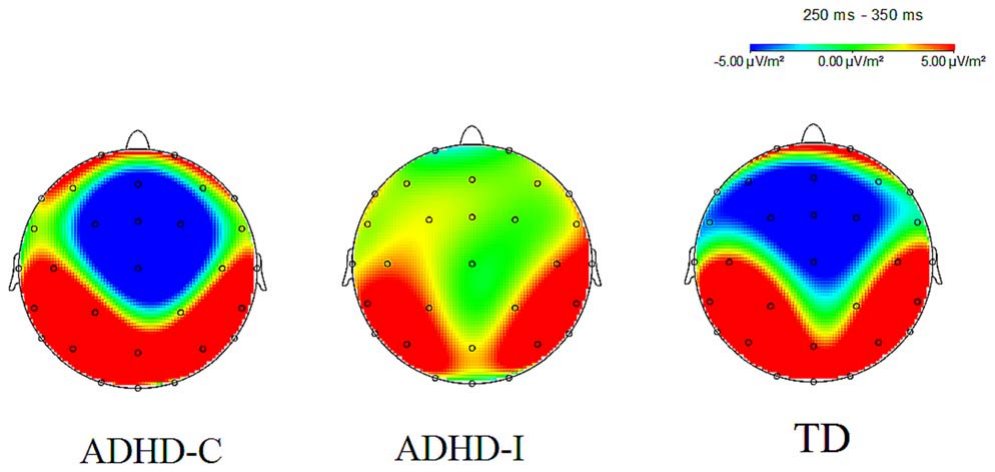
The topographical map of FRN in the TD,ADHD-C and ADHD-I groups in the phase of 250–350 ms.

The grand-average ERPs depicted in Figure 1. The topographical map of FRN in TD,ADHD-C and ADHD-I group was shown in Figure 2. The 3 (feedback type: large loss, small loss and gain feedback)×2 (electrode: Fz, Cz)×3 (group: TD, ADHD-C and ADHD-I participants) ANOVA revealed a significant effect of feedback type by group interaction effect during 250–350 ms [*F*(4, 86) = 2.49, *P* = 0.049]. When controlled for age and IQ score, the feedback type by group interaction effect was still marginally significant [*F*(4, 86) = 2.43, *P* = 0.054]. To further elucidate this interaction, we analyzed the feedback type effect in each group. In the TD group, the main effect of feedback type was significant [*F*(2, 28) = 4.16, *P* = 0.032], and large loss feedback elicited a larger negativity than did gain feedback [*F*(1, 14) = 10.11, *P* = 0.007] or small loss feedback [*F*(1, 14) = 4.34, *P* = 0.056], whereas no significant difference was observed between small loss and gain feedback [*F*(1, 14) = 0.59, *P* = 0.46]. In the ADHD-C group, the main effect of feedback type was significant [*F*(2, 30) = 4.88, *P* = 0.023], large loss feedback elicited a larger negativity than did small loss feedback [*F*(1, 15) = 19.29, *P* = 0.001]. However, no significant differences were observed between large loss and gain feedback [*F*(1, 15) = 0.1, *P* = 0.33], or between small loss and gain feedback [*F*(1, 15) = 3.09, *P* = 0.1]. In the ADHD-I group, no significant main effect of feedback type was observed [*F*(2, 28) = 0.91, *P* = 0.41]. In addition, we also broke down the feedback type by group interaction effect by analyzing the group effect during each feedback condition. No significant group effects observed during big loss feedback [*F*(2, 43) = 2.05, *P* = 0.14 ], small loss feedback [*F*(2, 43) = 0.03, *P* = 0.97 ], and gain feedback [*F*(2, 43) = 0.47, *P* = 0.63] conditions.

In order to analyze the large loss and small loss feedback effect, we further subtracted the gain feedback from large loss and small loss feedback. The 2 (feedback type: large loss vs gain, small loss vs gain)×2 (electrode: Fz, Cz)×3 (group: TD, ADHD-C and ADHD-I participants) ANOVA revealed a significant effect of feedback type by group interaction effect during 250–350 ms [*F*(2, 43) = 6.17, *P = *0.004]. To break down this interaction, we further analyzed the feedback type effect in each group, respectively. In the TD group, the main effect of feedback type was significant, and the large loss feedback effect is larger than small loss feedback effect [*F*(1, 14) = 10.11, *P* = 0.007]. In the ADHD-C group, the main effect of feedback type was significant, and the large loss feedback effect is larger than small loss feedback effect [*F*(1,14) = 19.29, *P* = 0.001]. In the ADHD-I group, no significant main effect of feedback type was observed [*F*(1, 14) = 0.34, *P* = 0.57].

In addition, the LPP component was also measured at 100 ms intervals during the 400–800 ms intervals at the Pz electrode site. When controlled for age and IQ score, the main effect of feedback type [*F*(2, 82) = 0.21, *P* = 0.8] and the feedback type by group interaction effect were not significant during 400–500 ms [*F*(4, 82) = 1.37, *P* = 0.25], but there was a significant main effect of group [*F*(2, 41) = 4.15, *P* = 0.023] during 400–500 ms. The LPP differences between the TD and ADHD-C [*F* (1, 27) = 2.69, *P* = 0.11], TD and ADHD-I [*F* (1, 26) = 0.39, *P* = 0.54] groups were not significant, but the LPP was larger in the ADHD-C group than in ADHD-I groups [*F* (1, 27) = 9.94, *P* = 0.004]. However, the main effect of feedback type [500–600 ms: *F*(2, 82) = 1.27, *P* = 0.29, 600–700 ms: *F*(2, 82) = 0.34, *P* = 0.69, 700–800 ms: *F*(2, 82) = 0.39, *P* = 0.66], group [500–600 ms: *F*(2, 41) = 2.18, *P* = 0.13, 600–700 ms: *F*(2, 41) = 0.72, *P* = 0.49, 700–800 ms: *F*(2, 41) = 0.59, *P* = 0.56] and the feedback type by group interaction effect [500–600 ms: *F*(4, 82) = 1.2, *P* = 0.32, 600–700 ms: *F*(4, 82) = 1.31, *P* = 0.27, 700–800 ms: *F*(4, 82) = 1.35, *P* = 0.26 ] were all non-significant during each 100 ms interval of the 500–800 ms intervals.

## Discussion

This is the first study to investigate the effect of feedback valence (reward or punishment) and punishment magnitude (small or large) on FRN and LPP in two subtypes of ADHD (ADHD-C and ADHD-I) compared to TD children during a children's gambling task. Analysis of the FRN interval revealed that TD children elicited a more negative FRN potential to large losses than to small losses or gains, which is consistent with previous studies showing larger FRN amplitudes to omitted gains and omitted losses in TD children [31], demonstrating that TD children exhibited enhanced monitoring of feedback when it was made more salient. These observations replicated prior findings that humans (including TD children) are especially sensitive to emotionally negative events, which are preferentially processed relative to neutral and positive events [49]–[51] and that humans are sensitive to magnitude differences in negative stimuli [56], [58], [64]. The children with ADHD-C also showed an enhanced FRN to large losses compared to that to small losses and gains, which is consistent with the finding of an enhanced sensitivity to unfavorable outcomes in children with ADHD [30], probably due to abnormalities in mesolimbic reward circuits. Affective evaluation and the assessment of future consequence of the feedback signal seems to be altered in ADHD. The children with ADHD-C did not differ in their feedback FRN amplitude from TD children, which suggests that children with ADHD are described as benefiting from renforcement contingencies from a clinical perspective. Reinforcement has proved to be highly effective in the treatment of ADHD [65] and that reinforcement contingencies are found to normalize behavior that characterizes ADHD [25]. Therefore, similar to TD children, children with ADHD-C are sensitive to punishment and magnitude differences in punishment. This finding may be explained by the fact that humans are equipped with a special sensitivity to negative stimuli as a result of evolutionary adaptation, as illustrated by the negative bias account [52], [56]. Another plausible explanation is that the motivational style of children with ADHD-C is characterized by competitiveness and a desire to be perceived as superior to others, which might result in enhanced sensitivity to negative outcomes [40], therefore, children with ADHD-C paid more attention to the larger losses. However, the FRN effect was absent under all conditions in children with ADHD-I, even under the large punishment condition, which contradicted the bias for emotionally negative events in humans. The absence of the FRN effect in children with ADHD-I may be explained by their nature of the cognitive-attention deficit, which is characterized as drowsy, sluggish and less alert [36], [37]. And compared to children with ADHD-C, the children with ADHD-I show different motivational style, possible less uncooperative and more passive in the learning style [38]. The FRN effect was elicited both in children with ADHD-C and in TD children, but not in children with ADHD-I, which may be attributed to the severe inattention problems, as the core symptom in children with ADHD-I, who suffer from a deficit detection of motivationally significant cues. However, one may question that both ADHD-C and ADHD-I showed similar symptoms of inattention, consequently this inattention explanation is probably unconvincing. It is worth note that ADHD-C children are characterized not only by inattention problems but also by competitiveness and perceived self-superiority. As discussed above, the latter characteristics are associated with enhanced sensitivity to negative outcomes which in turn enhances feedback negativity in brain potentials. This may constitute a compensating feature for ADHD-C children and explains why ADHD-C showed a robust FRN effect that was absent in ADHD-I, despite similar inattention problems in both groups. Also, this compensatory mechanism may explain why ADHD-C and TD children showed similar FRN effect, despite the inattention problems in the former group.

The present study observed an LPP component only in the early phase (400–500 ms) in children with ADHD-C, who showed an enhanced LPP amplitude to feedback indicating larger losses than TD children and children with ADHD-I. Furthermore, LPP has been reported to reach the largest amplitudes at 500–700 ms post-stimulus and to last for several hundred milliseconds [40]; In the current study, the LPP was larger in the ADHD-C group than in ADHD-I group [ *F* (1.27) = 9.94, *P* = 0.004]. This was consistent with a prior study by Carlson and colleague [38] implying that individuals with ADHD-C were motivated by competitiveness and desired to be suprior to others which might contribute to greater sensitivity to larger losses. However, LPP emerged only in the 400–500 ms interval rather than in a longer time epoch. Thus, this observation should be considered as tentative and awaits future studies to be replicated.

There are some limitations to the interpretation of the current findings. First, of our study was the relatively small sample size, especially for female participants. There was only one girl in the ADHD-I group, three girls in the ADHD-C group and four girls in the TD group, which did not make it possible to examine the potential role of sex. Second, in our study we only recruited children with two subtypes of ADHD (ADHD-I and ADHD-C), and not the hyperactive/impulsive subtype (ADHD-H). Thus, we could assess only differences between the ADHD-I and ADHD-C subtypes. Finally, the children's gambling paradigm used in the present study was designed only for one reward condition (+2), and two different punishment conditions (−4 or −1). We probably should have designed two reward conditions. Future studies are needed to investigate whether the same results can be obtained when reward and punishment have an equal number of conditions.

In conclusion, this is the first study to investigate to the effect of feedback valence and punishment magnitude on feedback negative and LPP in two subtypes of ADHD (ADHD-C and ADHD-I). In TD children and children with ADHD-C, large loss feedbacks evoked more negative FRN amplitudes than small loss feedbacks, suggesting that children with ADHD-C may keep intact brain sensitivity to the punishment and its magnitude. In contrast with the above two groups, the above FRN effect was absent in children with ADHD-I, suggesting that children with ADHD-I are significantly impaired in neural sensitivity to the feedback stimuli and in particular, to punishment. Secondly, the Late positive potential amplitudes were larger in children with ADHD-C in comparison with those with ADHD-I, regardless of feedback valence and magnitude. Therefore, we speculate that FRN, rather than LPP, is a reliable index to detect this difference across different ADHD-subcategories.
